# News and Coming Events

**Published:** 1907-03-16

**Authors:** 


### NEWS AND COMING EVENTS.

The Earl of Malmesbury has been re-elected President of the Royal Victoria Hospital at Bournemouth.

At the annual meeting of the Imperial Association of Austrian Physicians to be held in Vienna this year Professor Fraenkel will deliver an address on Lord Lister.

Professor C. Beadles (Hunterian Professor) will deliver a course of three lectures on "The Cerebral Arteries" at the Royal College of Surgeons, Lincoln's Inn Fields, on March 18, 20, and 22.

The annual meeting of the Kensington General Hospital, Richmond Road, S.W., will be held at the hospital on Saturday, March 16, at 3 p.m. Sir James Clark, Bart., will occupy the chair. A resolution will be tabled proposing the reduction of the representation of the medical staff from six to three members.

At the last monthly meeting of the Medical Sickness Annuity and Life Assurance Society the Secretary reported that the amount of sickness pay disbursed during the month of January last was the largest monthly amount ever paid by the society. This was largely due to the recent epidemic of influenza. The society has been in operation for twenty-three years, and many of its foundation members are now on half-pay, each member drawing 100 guineas a year.

Messrs. John Griffin and Sons, Limited, gave a Press and private exhibition at their new premises in Kingsway (corner of Kemble Street) on Wednesday last. The firm, founded in 1836 as a department for the sale of chemical apparatus, now deals in all kinds of scientific apparatus. The exhibition was interesting and instructive, and several demonstrations of novelties were given. Among those which attracted most attention were the latest models of the new Harcourt Chloroform Inhaler, the pharmaceutical machinery, including the most recent types of the tablet-making machine—a wonderful contrivance which mechanically compresses, polishes, and shapes 30,000 tablets an hour—the musical arc, the boy's calorimeter, and *x*-ray and wireless telegraph apparatus. One of the most popular rooms was the photographic room, where complete demonstrations of Goldona, Velox, and the Rawlins Pigment processes were given.

The annual meeting of the Association of Certificated Dispensers was held at Apothecaries' Hall on February 28. The Secretary said that the association had firmly established its position. He urged the necessity for members to maintain a high standard of efficiency in dispensing, and advocated a minimum age limit of twenty years and two years' training before granting a certificate, the acquisition of a distinctive title with the abolition of the title of "assistant," and a compulsory registration of members. The two Bills now before Parliament respecting the sale of poisons led him to suggest the advisability of the association promoting a Bill to give effect to the above propositions. The fact that the Government were now suggesting that certain poisons should be sold by totally unqualified persons opened up the question whether apothecaries' assistants should or should not be allowed to sell poisons when contained in medical prescriptions. At the same time, he thought the sale of poisons was a dubious privilege. Mr. Montagu Smith was elected Chairman for the ensuing year and Mr. A. L. Anderson Treasurer, Mr. Howell being re-elected as Secretary.

H.R.H. Princess Christian has given her name as the patroness of the Loughborough and District General Hospital.

The annual meeting of the Royal Eye Hospital, Southwark Street, will be held at the Hospital on Wednesday, March 20, at 4 p.m.

The annual meeting of the Royal National Hospital for Consumption will be held at the office, 34 Craven Street, Charing Cross, on Thursday, March 21, at 4.15 p.m.

A paper by Mr. Noel A. Humphreys, I.S.O., entitled "The Alleged Increase of Insanity," will be read before the Royal Statistical Society, at 9 Adelphi Terrace, Strand, W.C., on Tuesday, March 19, at 5 p.m.

Earl Cawdor has been elected President of the Alltymnod Sanatorium. Sir J. W. Drummond, Edwinsford; Sir Chas. Phillips, Haverfordwest; Colonel Davies-Evans, Highmead; and Mr. D. Davies, M.P., Plasdinam, were elected Vice-Presidents.

At the annual meeting of the Aldershot Hospital it was stated in the report that the enlargement of the institution is necessary. A children's ward is urgently needed, but before the Committee can definitely consider a rebuilding scheme the annual income for the upkeep of the hospital must be considerably augmented.

A Meeting will be held at the Mansion House on Wednesday, March 20, at 3 p.m., in order to promote the interests of the Second International Congress of School Hygiene to be held in London in August next. The Lord Mayor will preside, and among the speakers will be Sir Lauder Brunton, Sir Richard Martin, Bart., Dr. Macnamara, M.P., Sir John Cockburn, and Sir Edward Brabrook.

The committee of the Mission to Lepers has decided to erect three new asylums this year, and is appealing for funds to meet the expenses of the extension scheme. The mission already possesses 50 asylums, in which 4,000 lepers are being supported. Details of the scheme and of the work of the mission may be obtained from the Secretary, Exeter Hall, Strand.

One of the most interesting exhibits at the Olympia Motor Show is that of the New Leader Motors, Limited, of Nottingham. This company shows a business or professional man's car—a two-seater, with leather folding hood and folding screen. The body is of special design, built by Messrs. Brown and Hughes, of Shepherd's Bush. It is exceptionally roomy, with high side-doors, and may be said to be "weather-proof." It is fitted with drawers and boxes for papers and luggage, and wherein, it might be incidentally mentioned, a special compartment is provided for carrying the inevitable and inconvenient silk hat. An electric light is also fitted inside the hood. The engine is the Leader four-cylinder 10-12, and transmission is by cardan shaft, with the ordinary three speeds forward and reverse. The Leader firm claims to have achieved—by careful designing and the employment of the best labour and materials that can be obtained regardless of cost—in all their vehicles the following points—namely, reliability, efficiency, simplicity, accessibility, and silent running.

